# How Does an Online Patient-Nurse Communication Service Meet the Information Needs of Men with Recently Diagnosed Testicular Cancer?

**DOI:** 10.5402/2012/260975

**Published:** 2012-12-04

**Authors:** Torunn Wibe, Ragnhild Hellesø, Cecilie Varsi, Cornelia Ruland, Mirjam Ekstedt

**Affiliations:** ^1^Center for Shared Decision Making and Collaborative Care, Oslo University Hospital, Oslo Universitetssykehus HF, Medisinsk klinikk, Postboks 4950 Nydalen, 0424 Oslo, Norway; ^2^Department of Nursing Science, Institute of Health and Society, Faculty of Medicine, University of Oslo, Postboks 1130, Blindern, 0318 Oslo, Norway; ^3^Abildsø Nursing Home, Center for Development of Institutional Care Services in Oslo, Løvsetdalen 2, 1166 Oslo, Norway; ^4^School of Technic and Health, KTH Royal Institute of Technology, Alfred Nobels Allé 10, 14152 Huddinge, Sweden

## Abstract

Online communication has become a potential means of communication between patients and health care providers, but so far few studies are published about online communication as part of nursing care. The aim of this study was to explore how an online patient-nurse communication (OPNC) service meets the information needs of men with newly diagnosed testicular cancer. We applied a qualitative approach by examining the content of online messages sent by patients to nurses in a specialist cancer unit. In addition, individual interviews were conducted with patients who had used the OPNC service. Four themes became distinct through a synthesis of the material from the interviews and the messages: “a means for managing illness-related concerns at home,” “a means for ensuring information flow,” “a means for strategic information seeking,” and “not yet available when needed most.” Individualized information provided by nurses with access to their medical record was shown to be important to these patients. The findings of this study indicate that not only may access to an OPNC service help patients fulfill their otherwise unmet information needs, but also it may prevent delays and discontinuity in care due to informational gaps and lead to improved patient safety.

## 1. Introduction

In recent years, online communication has become one of the means by which patients can communicate with their health care providers, but its benefits have been disputed. Early studies included warnings that there might be some reduction in the quality of communication when using e-mail, since it entails the loss of nonverbal social cues that provide valuable contextual information in conversation [1]. Concerns have also been expressed that e-mail may reduce patient-provider communication to brief, task-specific electronic exchanges [2]. However, Roter et al. demonstrate that e-mail is an effective means of communicating health information, and that it can be a vehicle for providing emotional support as well as for building a therapeutic partnership. They conclude that the asynchronicity of place afforded by e-mail, with patients at home, may help to free patients from the social constraints of their role as a patient and enable them to convey sensitive, embarrassing, or especially distressing information that may be withheld in face-to-face visits [3]. 

Most studies of online communication between patients and staff in a healthcare setting have involved communication between patients and physicians [3–6]. To our knowledge, only a few studies so far have explored in depth patients' experiences of online communication with oncology nurses [7, 8]. In a recent study, Grimsbø et al. found that an online patient-nurse communication service helped to address the unmet information needs of patients with breast and prostate cancer, concerning issues such as symptoms, possible side effects of treatment, and results of blood tests, as well as fear of relapse and concerns for everyday life [9]. In that study, the nurses answering patients' questions did not have access to the patients' medical record or their individual medical history, which limited the extent to which they could provide information specifically relevant to that particular patient. We have found no similar studies of electronic communication experiences of patients with testicular cancer. In the current study, patients recently diagnosed with testicular cancer were offered access to an online patient-nurse communication service (OPNC). We also wanted to take a further step and to explore how patients would use the service if the cancer nurses answering questions were recruited from the hospital where the patients were receiving treatment and had access to the patients' medical record.

Testicular cancer is relatively rare, but it is the most commonly diagnosed malignancy in men aged between 15 and 40, with an increasing rate of incidence [10]. It is considered a highly curable form of cancer, but—like any other cancer diagnosis—it may have major physical and psychological effects for the individual. This patient group was considered especially appropriate for our study, because, as a cohort of young and middle-aged men, they represent a population segment with experience in using digital technology. Also, these individuals are in a busy phase of their lives, so the opportunity to pose questions to health care providers by e-mail may provide a useful supplement to other means of communication.

In Norway, where the current study took place, most patients with testicular cancer receive their diagnosis and initial surgical treatment at a local, secondary-level hospital. After the initial surgery, they are referred to a cancer specialist at a tertiary hospital for advanced investigations and for assessment of the need for further treatment. In cases where chemotherapy or radiation treatment is required, this is also normally performed at the tertiary hospital, whereas follow-up treatment takes place at the secondary care hospital or in primary care. Patients may find it difficult to identify to whom in the health care system they should be addressing their unanswered questions [9]. Access to an online communication service may help patients to obtain answers and some reassurance relating to the questions and concerns that arise between their visits to the tertiary hospital. 

The overall aim of the current study was to explore how an online patient-nurse communication service meets the information needs of men with newly diagnosed testicular cancer. Specific research questions were as follow.

- How do men with newly diagnosed testicular cancer use an online patient-nurse communication service?
- How do patients perceive that an online patient-nurse communication service meets their information needs in the early phases of a testicular cancer trajectory?

## 2. Methods

We selected a qualitative approach, because it allows for exploring variations in patients' experiences, in which we were interested [11]. We used an interpretive description methodology. This is an established qualitative method of inquiry that extracts elements from the social science tradition, to create forms of understanding important to the clinical field [12]. Interpretive descriptions often involve multiple data collection strategies to obtain comprehensive and contextualized interpretations of the phenomena of interest [13]. Completeness is important in a qualitative inquiry, as it allows for recognition of multiple realities [14]. We used triangulation, through the examination of messages and individual interviews, as a means of enlarging the landscape of our study, offering a deeper and more comprehensive picture [14].

In our study, we included *all *messages (*N* = 54) posted by patients with testicular cancer who had used an online patient-nurse communication (OPNC) service between November 2009 and April 2011. The messages had been sent by 12 different patients aged between 24 and 51 years (mean age 37, median 36). Each patient was offered access to the service for 8 months. The number of messages sent by each patient during that time varied from 1 to 11. In addition, we held face-to-face individual interviews with 5 of these patients, to obtain a more complete understanding of how the patients perceived that the OPNC service met their information needs.

### 2.1. The OPNC Service

The current study is a part of a larger study in which an OPNC service was implemented in a cancer department at a tertiary hospital in Norway. In the OPNC service, patients can communicate their questions and concerns and receive advice and support from cancer nurses in the hospital, between and after their hospital visits. Two expert cancer nurses, experienced in the care and treatment of patients with testicular cancer, were selected by the specialty oncology unit to manage the OPNC service and consented to contribute to the study. The nurses had access to the patients' medical records and answered the patients' questions and concerns within two days during regular week day hours. The service allowed for patients' questions to be forwarded to physicians and social workers.

The Norwegian government requires a high level of internet security with strong authentication to enable patients to communicate personal health information on the internet. Messages in the OPNC service were sent using an encrypted connection. At the hospital, the service was integrated into the secure hospital network. To log on, patients receive a password or personal identification number (PIN), and a disconnected security token in the form of a physical device that generates a one-time authentication code for online identification. This is the “Bank ID” system currently used for Internet banking in Norway, so it is well-known to most Norwegians. 

### 2.2. Study Participants

The patients eligible to take part in the study were those who within the previous three months had been diagnosed with testicular cancer and who were undergoing treatment in the specialist cancer unit where the study was located. They had to be over the age of 18 and able to speak and read Norwegian. They also had to have an internet connection with secure access (Bank ID) at home and to have the technical confidence to enter text in the OPNC system with the help of a user's manual. 

### 2.3. Procedures

 At patients' first visit to the tertiary hospital, a nurse in the cancer department provided information to patients who met criteria for participation, informing them about the study and that they were eligible for participation, if interested. At that point, patients had already received initial surgical treatment at a local, secondary hospital, and they had been directed to the specialist unit for assessment of further treatment. Those interested in hearing more about the study were referred to the project assistant, who explained the purpose of the study and the use of the OPNC service, collected informed consent, and filled out the necessary registration forms. 

Some of the patients who took part in the study were selected for interviews, in May and June 2010. We applied convenience sampling and chose to select for interview only patients who lived in the eastern part of Norway, within 150 km (93 miles) of the hospital where the study took place, so as to make the most of the limited time and travel budget available for the study. The first author conducted individual interviews. An interview guide was used, which focused on the participants' experiences of their information needs and their access to information in general during their illness trajectory, as well as on their experience of using the OPNC service for information purposes. Thus, the interviews contributed data concerning the participants' experiences, which complemented the information about the actual use of the service and the content of the messages which was obtained from analysis of the messages. The interviews were recorded using a digital voice recorder, and they were transcribed verbatim. After five interviews, we found that they no longer provided any new information concerning our research questions. At that point we judged that the five interviews, together with the 54 messages, formed a large enough body of material, providing sufficient variety of experiences, to fulfill the aims of the study. 

### 2.4. Ethical Considerations

The study design was approved by the Norwegian Regional Research Ethics Committee, and it was supported by the Data Protection Official at the hospital. Study participants were asked to provide their written consent to participate in the study, including giving permission for the research team to access their messages in the OPNC service for research purposes.

### 2.5. Analysis

All of the messages from each of the patients who used the service were harvested from the server with the help of the system support team. Names and other personally identifiable information were deleted before the messages were copied into a Microsoft word file for analysis. 

Drawing on interpretive description methodology, the first author initially read the interview transcripts several times, to get a sense of the whole, beyond the immediate impression of what the interviews contained. Next, open coding was applied to identify recurring, converging, and contradictory themes in the text. Once this had been done, the available options for grouping the data were discussed with the research team, and a preliminary description of thematic linkages was prepared.

In the next step, the individual messages were analyzed in the same way; each message was initially read to gain an understanding of the inherent meaning of the message. Meaning units were then identified and condensed into codes. By moving back and forth between the parts and the whole, we were able to synthesize meaning units and codes from both interviews and messages, and once again a meaningful shared understanding was reached. On the basis of this analysis, we have generated an interpretive description of (1) how patients with testicular cancer used the online patient-nurse communication service and (2) how well in their perception this service met their information needs.

## 3. Findings

When we focused on the first research question in the analysis, the patients' *use* of the OPNC service was consolidated into two themes: “a means for managing illness-related concerns at home” and “a means for ensuring information flow.” These themes became distinct through synthesizing material from both interviews and messages. Two themes, “a means for strategic information seeking” and “not yet available when needed most” emerged when we focused on the second research question, about how patients *perceived* that the OPNC service met their information needs. These themes are presented below, illustrated by quotations from the interviews and messages to increase the transparency of the interpretation.

### 3.1. A Means for Managing Illness-Related Concerns at Home

This theme indicates that the questions addressed through the OPNC service formed a continuum from existential to practical issues. The patients used the OPNC service to take action about concerns and uncertainty relating to cancer symptoms, test results, side effects of treatment and possible metastases, as shown in the following message.

*“I was at a 4 weeks' check-up with you last week. Took some blood tests then, which showed some values that were too high. I was originally supposed to come back to you after 6 weeks, but now I've got a new appointment already after 2 weeks. How should I interpret this—a bigger chance that there is something in my body? Many people say that these values on blood tests go up and down. So I am very unsure what to think. Can I say that I don't have cancer any more now, until something more is discovered?” (message)*

Another patient expressed some concerns in a message due to continued high creatinine values in the blood after chemotherapy. The patient himself did not understand that this might be a sign of serious kidney failure, but his message led the tertiary hospital staff to take quick action to save the patient's kidneys from permanent damage.

Study participants expressed that it was not easy to remember to ask all the questions they had when they were at the hospital. New and unforeseen questions and concerns often came up when they were at home, between hospital appointments. In such situations, both telephone communication and the OPNC service were experienced as helpful in reducing uncertainty. It appears that both individual and situational factors affected the patients' decisions about whether to choose to use the telephone or OPNC as a means of communication with the hospital staff. Some study participants said that they were selective in their use of e-mail communication, knowing that several health care providers at the unit might have access to read their messages. Others explained that there were some questions they actively preferred to ask through the OPNC service rather than in direct contact with health care providers or on the telephone as follow.

*Sexual questions for example, which might have come up during the doctors' rounds … This might be easier to ask about in an e-mail to a person that you don't know than when the doctor asks: “What about your … (sexual function)?” Then you answer: “Oh, that's OK” or “That's normal” or whatever … (interview)*

There were also instances when patients had been met in an unfriendly or not very helpful way in a telephone call and then used the OPNC service to bypass the obstacle they met on the telephone as follow.

*Hi, I've been trying to get in contact with Dr. X since I last was in the hospital, but nobody would put me through and nobody would leave a message—so now I am trying to get through here … (message)*

### 3.2. A Means for Ensuring Information Flow

This theme illustrates how patients use the OPNC service for reassurance about information flow concerning follow-up care, which often involves cooperation between different levels of health care providers. One patient explained that, through the answer from a nurse to one of his e-mailed questions on the OPNC service, he had discovered that he was expected to come to the hospital two days later. Unfortunately, he had not yet received any information about his appointment through the usual channels. Other patients wanted to verify that the time scheduled for their check-up appointment at the hospital was realistic, as several departments at the hospital were involved, for instance,

*Hi. I got a letter yesterday about an X-ray examination on the 7th of March at 09.30. This is the same day that I have a consultation with the physician at 08.30. I figure that I will also go to the laboratory for a blood test that morning, before the consultation. I just want to make sure that the consultation will not conflict with the X-ray. If I have to wait for the consultation I risk arriving too late for the X-ray appointment. (message)*

Another patient wrote in a message that he was uncertain whether his primary care physician would know which blood test he needed and where to send it. Other patients sent a message because they had simply lost or forgotten the exact time of their next appointment at the hospital or because they were afraid that the hospital had forgotten to notify them of the appointment. 

Patients expressed uncertainty about whether the staff at their local secondary hospital or the primary care physician had received the appropriate information from the tertiary hospital so as to be able to provide the necessary follow-up care after treatment. They used the OPNC service for reassurance about this issue.

### 3.3. A Means for Strategic Information Seeking

In the interviews, patients described how the OPNC had become an important source of individualized information for them. They explained how, at an early stage, they decided to focus on getting well and becoming “expatients” as soon as possible. They did not want to dwell more than strictly necessary on their illness but quickly limited their interest to “my case” or “what is relevant for me”. Because of their preference for information about their particular case, individualized information given to them by health care providers who had access to details about their particular situation appeared to be of central importance for these patients. They described how, to a greater or lesser extent, they had searched for information on the internet during the waiting period after the initial surgery. However, because the details about their situation were not yet clear, it was often difficult to discern what information was relevant for their particular situation. One interviewee commented as follow.

*… It (the information on the Internet) was a little contradictory … […] It said that there are two types (of testicular cancer): there is non-seminoma and there is another type. And one of them is bad and the other is not that bad. But as long as you don't know what you have, it is really better not to read it, because … I at least, became more worried. You start to think about the worst case, you know … (interview)*

Study participants expressed that general cancer information on the Internet did not meet their information needs in the early phase of their illness trajectory. They wanted more individualized information, preferably given to them by health care providers who knew their case. They perceived that having access to the OPNC service once they had been referred to the tertiary hospital provided an additional source of individualized information and support. The analysis showed that having the opportunity to choose what kind of issues to discuss in direct contact sessions or by means of telephone or through the OPNC increased the patients' sense of having a degree of control over their situation, as illustrated below:

*… The combination, with good care when you meet in the hospital or speak on telephone, and in addition this OPNC service … This helped me a lot. (interview)*

### 3.4. Not Yet Available When Needed Most

The analysis of the interviews showed that the participants experienced an information vacuum from the point when they had undergone their first surgery at the local secondary hospital until the findings from tests and biopsies were available, and they knew whether they had to undergo radiation treatment or chemotherapy. Surgery was performed at the local secondary-care hospital within a couple of days after they had been informed that they had testicular cancer. At that stage, patients perceived they had been given priority and that they were well informed at this point of their illness trajectory. The decision about further treatment would, however, be taken in the cancer department at the tertiary hospital, to which they were referred after the initial surgery. During this waiting period, they felt that they had been left in an information vacuum where they had no one to turn to. Their primary care physician, who had the formal responsibility for their care, was not necessarily able to meet the patients' information needs. When they eventually gained access to the OPNC service, they recognized that this could have been a helpful resource while they were waiting for admission to the tertiary hospital.

*It's usually at the start of an illness that you have questions, and perhaps some extra need for support […] … Like after my first surgery at the local hospital and before I was admitted to the regional hospital—who could I talk to in the meantime?” […] “So my only complaint about the OPNC service is that it should have been accessible sooner after the (initial) surgery. (interview)*

Patients described this wait as a frustrating period, but they knew that at this point no one could give them definitive answers about what additional treatment they might need or about the likely course of their illness. One study participant said the following:

*… Yes, there was much that was still not clear, but on the other hand it would have been better to hear that “This is not clear yet, so you will have to wait until we have an answer to this and that before we can tell you”, rather than just: “We cannot answer you”. (interview)*

## 4. Discussion

In line with findings from similar studies on patients with other cancer illnesses [7–9], this study shows that an online patient-nurse communication service has great potential for helping to meet patients' information needs in the early phase of their illness trajectory and to increase patients' involvement in their own care. What the current study adds is demonstrating the function of an online service as a complementary source of individualized information, in helping patients to get exactly the information they need, at the time when they need it. Further, this tool was identified to be useful for the patients to ensure the necessary flow of information between different providers involved in their care.

### 4.1. Obtaining the Information You Need at the Time You Need It

The exploration of *how patients used* the OPNC service showed that patients made active use of the opportunity provided by the service to ask clarifying questions related to their illness and medical treatment. Earlier studies have shown that patients often find it difficult to identify relevant questions prior to discharge and that illness-related questions and concerns often arise once they are at home [15]. In this study, the OPNC service provided answers to questions that arose between hospital visits. Some participants also said that they dared to pose questions by means of the OPNC service that they would not have asked in an ordinary consultation (e.g., questions about sexuality). This phenomenon has also been found in other studies [4, 6]. At the same time, some study participants said that they were selective in their use of e-mail communication, knowing that several health care providers at the unit might have access to their messages. This finding also corresponds with findings in other studies [7].

The OPNC service provides the patient with written answers to his questions and the facility to go back and read the information again, which is not possible for information provided in face-to-face encounters or by telephone. With the distress and concerns associated with many of the issues raised in the questions posed by the patients, the opportunity to read the answers several times is valuable to the patients. This may also save health care providers from answering the same questions repeatedly. 

It is well known from studies in acute care that patients' information needs vary along the illness trajectory [15, 16] and that *the timing* of information provision is crucial for patients' ability to absorb and make sense of the information [15]. Having access to different means of communication helps patients to get the information they need, at the time when they need it, and at the time when they are ready to receive it. This can reduce their feeling of being at the mercy of health care providers' schedules and increases their feeling of control [17]. At the same time, it increases the chance that the information given is actually understood. 

### 4.2. Contributing to Health Care Effectiveness

Patients in our study valued the OPNC nurses' ability to provide more specific answers to their questions, due to their access to the patients' medical records. The fact that the nurses answering the patients' questions were working in the cancer unit where the patients received treatment also made it possible for patients to use the OPNC service to check details concerning their appointments at the hospital. So the messaging system helps to prevent delays and discontinuity in care due to problems such as patients not showing up for appointments, tests not taken or taken unnecessarily, or missing referrals. This aspect of the service was not included in the questions addressed in an earlier, comparable, OPNC study in Norway [9]. However, this was perceived an important benefit of the service and should therefore be considered when planning the scope of future OPNC services. 

In our study, the OPNC service was found to have an important role in situations when patients experienced a sense of insecurity about information transmission. Patient initiatives to ensure information flow between different providers are also described in other studies [18]. By offering direct contact with the specialist unit in the tertiary hospital, the OPNC service can contribute to reassuring the patient that the staff of the local, secondary hospital, or the primary care physician has sufficient information and knowledge to take care of him. However, the findings of the current study also demonstrate that ensuring good communication between different entities should be a high priority within the cancer care organization as well as in health care legislation.

### 4.3. A Comfortable Level of Knowledge

The issue about how the study participants perceived that the OPNC service met their information needs should be seen in relation to their information-seeking strategy. Participants in our study expressed limited interest in obtaining general information about their illness, but they searched for specific information about their individual situation. This is consistent with what Lambert et al. [19] describe as *complementary information seeking*—the process of acquiring sufficient information about their condition and wanting to reach a satisfactory level of knowledge about what is going on. Lambert et al. suggest that information behavior may go beyond the simple dichotomy of seeking and avoiding. They found that patients' information seeking varied along a continuum from intense information seeking to guarded information seeking. Complementary information seeking is characterized by purposefully seeking cancer information that is useful to understand what was going on *at a specific point in time *(pacing) [19]. This approach to managing cancer information is consistent with the findings in the current study. The participants explained that they had no wish to dwell unnecessarily on details about the illness, but that they wanted to receive the appropriate treatment and to carry on with their lives. A similar, common attitude towards their illness has also been described by other patients with testicular cancer: “I intended to treat it like a broken bone—get it fixed and carry on” [20]. This attitude toward their illness obviously influenced our study participants' information-seeking strategy, and the OPNC service was very responsive to that strategy, by providing patients with individually tailored information when it was requested.

### 4.4. An Information Vacuum at Particular Points of the Illness Trajectory

Although participants were very satisfied with the information and support that they received initially at the local, secondary hospital, they experienced an information vacuum in the period between the initial surgery at the local hospital and their first appointment at the specialist cancer unit at the tertiary hospital. Gaining access to the OPNC service at the tertiary hospital was obviously a positive experience for the study participants, as it made the health care professionals more accessible for responding to their questions and concerns [17]. However, the patients wished they had access to this service earlier. 

For cancer patients, waiting and uncertainty about diagnosis, treatment, remission, and possible relapse are often described as the worst part of the illness experience [17]. Although waiting and uncertainty are unavoidable in the early stages of treatment, as test results are still not available, unnecessary waiting time and unanswered questions should be avoided in order to reduce patients' feeling of powerlessness. We suggest that, at that stage of the illness trajectory, patients would find it helpful to be able to communicate with an expert nurse or physician who has access to information about their particular case, even if exact answers cannot be given to all questions. The provision of a better information service such as an OPNC service for patients who are waiting for admission to the tertiary hospital appears to be a potentially helpful way of improving cancer care. 

### 4.5. Methodological Considerations

This study has certain limitations. The size of the sample is relatively small, and we therefore used data triangulation aiming to strengthen credibility. We regard the assembled material, consisting of 54 available messages, together with the 5 interviews, as large enough to yield a sufficient variety of patient experiences to provide useful insights, in line with interpretive description methodology [13].

Another limitation may be that the study participants knew that their messages might be read by the researchers for research purposes, although anonymously, and also by other members of the health care team who had access to the service. Some of these patients may have had this “larger audience” in mind when formulating their questions, perhaps withholding questions that they might have asked if the OPNC nurse had been the only person reading them. 

A rigorous analytic process in interpretive description involves inductive reasoning, including testing and challenging preliminary interpretations, and conceptualizing an ordered and coherent final product [13]. In line with this, and to address trustworthiness, available options for grouping the data were discussed with the research team. Preliminary descriptions of thematic linkages were prepared and revised several times until a common understanding was reached. Interpretive description acknowledges the constructed and contextual nature of human experience that at the same time allows for shared realities [13]. Thus, the aim of the discussions in the research team was not to reach consensus but to broaden and find alternative interpretations and the best ways to present the findings. 

## 5. Conclusions

This study demonstrates that an online patient-nurse communication service has the potential to help address patients' unmet information needs and so to increase patients' feeling of control. Participants in this study said that they wished they had been offered access to such a service earlier in their illness trajectory. The study participants used the OPNC service as a means for managing illness-related questions and concerns as well as for helping to ensure the necessary flow of information between different providers involved in their care. Increasing patients' opportunity to participate in the management of their treatment program by means of an OPNC service may thus prevent delays and discontinuity in care due to informational gaps and lead to improved patient safety.
